# Faster Serotonin Clearance in CA3 Region of Hippocampus and Antidepressant-like Effect of Decynium-22 in Juvenile Mice Are Putatively Linked to Increased Plasma Membrane Monoamine Transporter Function: Implications for Efficacy of Antidepressants in Juveniles

**DOI:** 10.3390/cells11152454

**Published:** 2022-08-08

**Authors:** Melodi A. Bowman, Jorge A. Gomez, Nathan C. Mitchell, Anne M. Wells, Melissa Vitela, Kyra M. Clarke, Rebecca E. Horton, Wouter Koek, Lynette C. Daws

**Affiliations:** 1 Department of Cellular and Integrative Physiology, University of Texas Health Science Center at San Antonio, MC7756, 7703 Floyd Curl Drive, San Antonio, TX 78229-3900, USA; 2 Department of Cell Systems and Anatomy, University of Texas Health Science Center, San Antonio, TX 78229-3900, USA; 3 Department of Pharmacology, University of Texas Health Science Center, San Antonio, TX 78229-3900, USA

**Keywords:** antidepressant, decynium-22, depression, juvenile, organic cation transporter 3, plasma membrane monoamine transporter, serotonin clearance, serotonin transporter

## Abstract

Selective serotonin reuptake inhibitors (SSRIs) are less efficacious in treating depression in children than in adults. SSRIs block serotonin uptake via the high-affinity, low-capacity serotonin transporter. However, the low-affinity, high-capacity organic cation transporter 3 (OCT3) and plasma membrane monoamine transporter (PMAT) are emerging as important players in serotonin uptake. We hypothesized that OCT3 and/or PMAT are functionally upregulated in juveniles, thereby buffering SSRIs’ ability to enhance serotonergic neurotransmission. Unlike in adult mice, we found the OCT/PMAT blocker, decynium-22, to have standalone antidepressant-like effects in juveniles. Using in vivo high-speed chronoamperometry, we found that juveniles clear serotonin from the CA3 region of the hippocampus ~2-fold faster than adult mice. Cell density did not differ between ages, suggesting that faster serotonin clearance in juveniles is unrelated to faster diffusion through the extracellular matrix. Western blot and immunohistochemistry showed that juvenile mice have modestly greater expression of PMAT than adults, whereas OCT3 expression in the CA3 region of the hippocampus was similar between ages. Together, these data suggest that faster serotonin clearance and antidepressant-like effects of decynium-22 in juvenile mice may be due to functionally upregulated PMAT. Faster serotonin clearance via PMAT in juveniles may contribute to reduced therapeutic efficacy of SSRIs in children relative to adults.

## 1. Introduction

Depression affects individuals of all ages; however, medications to treat depression are less effective in children and adolescents than in adults [1]. Fluoxetine and escitalopram are the only antidepressants approved by the Food and Drug Administration (FDA) for use in individuals under the age of 18. Both antidepressants belong to the selective serotonin reuptake inhibitor (SSRI) class of antidepressants, which work by blocking the serotonin transporter (SERT). SERT is the high-affinity uptake mechanism for serotonin; however, it has a low capacity for serotonin uptake. Blockade of SERT by SSRIs prevents uptake of serotonin from extracellular fluid, and the subsequent accumulation of serotonin is thought to trigger neural events that ultimately bring relief of depressive symptoms. Since SSRIs are less effective in children than in adults, this raises the possibility that in juveniles and adolescents, there may be another mechanism(s), or transporter(s), at play that prevents accumulation of serotonin in extracellular fluid.

Organic cation transporter 3 (OCT3) and plasma membrane monoamine transporter (PMAT) are low-affinity, but high-capacity transporters of serotonin [2,3,4,5,6,7,8,9,10,11,12,13,14]. We have shown that a sub-effective dose of the SSRI fluvoxamine, in conjunction with decynium-22 (D22, a blocker of OCTs and PMAT), which by itself does not have antidepressant-like effects, produces robust antidepressant-like effects in adult mice [15]. We also found an antidepressant-like effect of D22 in adult mice with a 50% reduction in SERT [SERT heterozygous (+/−) mice] or lacking SERT [SERT knockout (-/-) mice], which may be driven by increased OCT3 expression in these genotypes [16]. Thus, it is possible that OCT3 (and/or PMAT) expression and/or function is upregulated in juveniles/adolescents, thereby decreasing efficacy of SSRIs to treat depression in children/teenagers.

To our knowledge, there have not been any studies examining the ontogeny of OCTs or PMAT in brain. However, OCT1 mRNA expression reaches adult levels by (postnatal day, P) P15 in kidney and by P22 in liver [17]. Although it is difficult to generalize these findings to changes that may occur in brain, these data support the idea that in juveniles, OCTs, putatively OCT3, and PMAT may already be at adult levels, or perhaps transiently expressed at higher levels than in adults. If so, these ancillary serotonin clearance mechanisms may undermine the therapeutic utility of drugs that selectivity block SERT (SSRIs) in juveniles.

The goals of the present study were to evaluate the antidepressant-like effects of D22, assess functional clearance of serotonin in vivo and quantify expression of OCT3 and PMAT in juvenile P21 and adult (P90) mice. Although D22 has activity at all three OCT subtypes (OCT1, OCT2 and OCT3) and PMAT, we focused on OCT3 and PMAT, and not OCT1 or OCT2, because D22 is generally reported to have higher affinity for OCT3 and PMAT compared to OCT1 and OCT2, and monoamine uptake by OCT1 is very limited [18,19]. Importantly, D22 lacks affinity at SERT, the dopamine transporter (DAT) and the norepinephrine transporter (NET) [19]. We found that D22 exerts an antidepressant-like effect in juvenile, but not adult mice; that serotonin clearance is ~2-fold faster in juveniles than adult mice, and that this corresponds to modestly greater PMAT expression in juvenile mice than in adults, with no apparent difference between ages in cell density, suggesting that faster clearance is unlikely due to lower tortuosity in juvenile mouse brain than in adults. Taken together, results from this study suggest that juveniles transiently express higher levels of functional PMAT than adults, which, in turn, could account for reduced efficacy of SSRIs in children.

## 2. Materials and Methods

### 2.1. Animals

Naïve male postnatal day 21 (P21, juvenile) and P90 (±3 days, adult) C57BL/6 (for tail suspension test (TST) studies), male SERT wild-type (+/+) mice bred on a C57BL/6 background (for western blot) or male PMAT+/+ mice bred on a C57BL/6 background (for all other studies) were used. We selected these ages because in rodents, P21-27 is considered the juvenile period, while greater than P60 is considered adult [20]. Mice were housed in plastic cages (29 cm × 18 cm × 13 cm) containing 7090 Teklad sani-chip bedding (Envigo, East Millstone, NJ, USA) and maintained on a 12/12-h light/dark cycle (lights on at 7:00 am) in a temperature-controlled (24 °C) vivarium. Mice were weaned at P21 and housed with same sex littermates with no more than 5 mice per cage. Mice were given free access to food (Teklad LM-485 mouse/rat sterilizable diet 7012 chow [Envigo, East Millstone, NJ, USA]) and water. All procedures were conducted in accordance with the National Institute of Health Guide for the Care and Use of Laboratory Animals (Institute of Laboratory Animal, Resources, Commission of Life Sciences, National Research Council, https://grants.nih.gov/grants/olaw/Guide-for-the-Care-and-use-of-laboratory-animals.pdf, accessed on 15 December 2014, and intermittently thereafter), and with the approval of the Institutional Animal Care and Use Committee, The University of Texas Health Science Center at San Antonio.

### 2.2. Tail Suspension Test

The tail suspension test (TST) was conducted as previously described in Steru et al. ([21], and see [22,23] for reviews) to assess antidepressant-like effects. All experiments were conducted between 12:00 pm and 5:00 pm by the same male experimenter. Mice were moved from the colony room to the behavior testing room approximately 1–2 h before starting the experiment. Mice received either saline or 0.1 mg/kg D22 intraperitoneally (i.p.) 1 hour before testing (injection volume 10 mL/kg). This dose of D22 was chosen based on previous studies from our lab examining the ability of D22 to enhance the antidepressant-like effects of SSRIs [15] and produce antidepressant-like effects in SERT mutant mice [16]. This dose of D22 does not affect locomotor activity. Mice were randomly assigned to treatment conditions. To initiate testing, the distal portion of the mouse’s tail was fastened to a flat aluminum bar (2 cm × 0.3 cm × 10 cm) using adhesive tape leaving 2–3 cm (P21) and 3–4 cm (P90) between the base of the tail and aluminum bar. A hole in the end of the aluminum bar was placed on a hook on the ceiling of a visually isolated white box (40 cm × 40 cm × 40 cm) to suspend the mouse during the TST. Once the aluminum bar attached to the mouse was secured to the hook, the mouse was rotated so that its ventral surface and all four paws were facing the video camera used to record the mouse’s movements during the 6 min test. After the test, the aluminum bar was taken off the hook, and the mouse was removed from the aluminum bar and placed back into a transportation cage. All mice were naïve before TST and each mouse was only tested once. Videos were scored by individuals blinded to treatment.

### 2.3. High-Speed Chronoamperometry

In vivo high-speed chronoamperometry was used to examine serotonin clearance kinetics in P21 and P90 wild-type mice. The same individual (a female) performed all experiments. Experiments were conducted using methods adapted from our previous publications (e.g., [15,16,24,25]). Sensitivity to serotonin and its metabolite 5-hydroxyindoleacetic acid (5-HIAA) were measured by calibrating Nafion-coated electrodes to increasing concentrations of serotonin (0.2 to 1.6 µM in 0.2 µM increments) in the presence of 5-HIAA (250 µM). Only those electrodes with a selectivity ratio for serotonin over 5-HIAA greater than 100:1 and a linear response (r^2^ ≥ 0.9) to serotonin were used.

Barrels of the micropipette were filled with serotonin (200 µm) in phosphate-buffered saline (PBS). The electrode-micropipette assembly was lowered into the CA3 region of the hippocampus (adult mouse: anteroposterior, −1.93 and mediolateral +2.0 from bregma; -2.0 from dura [26] of an anesthetized mouse. Minor adjustments were made to stereotaxic coordinates for juvenile mice. Since a brain atlas of stereotaxic coordinates does not yet exist for P21 mice, these coordinates were determined empirically. The stereotaxic coordinates used for CA3 region of hippocampus in P21 mice were: anteroposterior: -1.8 and mediolateral +2.0 from bregma; −1.8 from dura. Isoflurane (5%) was used to initially anesthetize the mouse and 1.0–1.5% isoflurane was used throughout the experiment to maintain anesthesia. Body temperature was maintained at 36–37 °C by a water circulated heating pad.

The FAST-16 system (Quanteon, Nicholasville, KY, USA) was used for high-speed chronoamperometric recordings. Exogenous serotonin was pressure ejected into the CA3 region of hippocampus. Serotonin was pressure ejected to achieve signal amplitudes that ranged from ~0.25 to 1.5 µM. Serotonin clearance rate (T_c_; the rate at which serotonin is cleared from the extracellular fluid over the pseudo-linear descending phase of the signal, i.e., between 20% and 60% of the peak signal amplitude), T_80_ time course (the time it takes for the signal to decline by 80% of the peak signal amplitude), and T_20_–T_60_ time course (the time it takes for the signal to decline by 20–60% of the peak signal amplitude) were analyzed.

At the completion of the experiment, an electrolytic lesion was made to mark the placement of the electrode tip. Brains were removed, frozen and stored at −80 °C for histological analysis. Brains were thawed to −18 °C and sliced into 20 µm thick sections and stained with thionin (a stain specific for DNA and Nissl substance, which is primarily ribosomal RNA) for verification of electrode placement and cell density analyses.

### 2.4. Cell Density

Brains from P21 and P90 mice used in chronoamperometry experiments were used to examine cell density. Since the electrode for chronoamperometry experiments was placed on the right side, the left CA3 region of hippocampus was examined. Photomicrographs were taken of three representative sections through CA3 region of hippocampus in brightfield for maps (120× magnification) and cell counts (300× magnification) using an Olympus microscope and a CCD camera connected to a commercial imaging system (QCapture version 3.1.1).

To count the number of neurons and microglia present in the CA3 region of the hippocampus of thionin-stained brain sections, an algorithm adapted from primates [27] and applied to mice [28] was used. Neurons and microglia were identified and differentiated from each other by morphological features highlighted by thionin staining. The most common type of neuron in CA3 region of hippocampus are pyramidal and were identified by visible cytoplasm around the nucleus and a darkly stained nucleolus. Microglia were identified by ovoid, elongated or comma-shaped nuclei without visible cytoplasm.

Maps of the CA3 region of the hippocampus of each mouse were created to aide in cell counting. Photomicrographs were imported into Adobe Illustrator, boundaries of CA3 region of hippocampus were traced and each neuron and microglia was marked. The program then counted the number of “marks” to determine the number of neurons and microglia in each section.

The area of the CA3 region of the hippocampus was measured to determine whether there were differences in size in the CA3 region of the hippocampus between juvenile and adult mice. The same thionin-stained brain sections used for cell counting were used for this analysis. The photomicrographs were imported into ImageJ and boundaries of CA3 region of hippocampus were traced. The program measured the area within the trace.

### 2.5. Western Blot

Mice were euthanized by rapid decapitation, brains removed and hippocampi dissected out on ice and stored at −80 °C until use. Samples were suspended in 500 µL of ice cold PBS (0.1 M, pH 7.4) and pulsed in centrifuge. Supernatant was removed, and pellets were then homogenized in 500 µL of homogenizing buffer (25 mM Hepes, 25 mM sucrose, 1.5 mM MgCl 2, 50 mM NaCl, pH 7.2, aprotinin, leupeptin, pepstatin, PMSF) by using glass Potter-Elvehjem tissue grinders. Total protein concentrations of each sample were measured by Bradford Protein Assay [29]. Samples were solubilized in a buffer containing bromophenyl blue, heated for 5 min at 95 °C and separated by electrophoresis using SDS-polyacrylamide gel electrophoresis (SDS/PAGE). Protein was then transferred to a polyvinylidene difluoride (PVDF) membrane (Immobilon-P, Millipore). The membrane was blocked in 5% nonfat dry milk in 1 TBS-Tween at room temperature for 1 h, washed twice with 1 TBS-Tween and incubated overnight at 4 °C with either polyclonal OCT3 antibody (Alpha Diagnostics International, San Antonio, TX, USA), monoclonal SLC29A4 (PMAT) antibody (Abcam, Cambridge, MA, USA) or monoclonal actin antibody (Sigma-Aldrich, St Louis, MO, USA). The primary antibody was removed and the membrane was washed 3 times and incubated for 1 h at 4 °C with the secondary ECL donkey anti-rabbit IgG horseradish peroxidase-linked antibody (1:2500) (GE Healthcare) or secondary ECL sheep anti-mouse IgG horseradish peroxidase-linked antibody (1:2500 for SLC or 1:5000 for actin). Bound antibody was detected on x-ray film by using enhanced reagents (GE Healthcare). The same blot was probed with all 3 antibodies for comparison. SDS-stripping was performed in between each probing.

### 2.6. Immunohistochemistry

Naïve mice were anesthetized and intracardially perfused with ice cold PBS, followed by 4% paraformaldehyde (PFA). Brains were removed and placed in 4% PFA overnight at 4 °C. Brains were then rinsed with PBS, and 50 µM coronal sections containing CA3 were collected. Sections were placed in PBS at 4 °C.

Sections were rinsed three times with PBS for 5 min, and then incubated in 0.05% sodium borohydride-PBS (Sigma-Aldrich, St Louis, MO, USA, 452884) for 30 min. Sections were then blocked using 5% normal goat serum (NGS, ab7481) in PBS for 1.5 h at room temperature. Sections were incubated for 3 days at 4 °C with either of the following primary antibodies: Rabbit anti-OCT3 (1:100, OCT31A Alpha Diagnostics International) or rabbit anti-PMAT (1:250, bs-4176R) in 5% NGS PBS. On day four, sections were rinsed 3 times for 5 min with PBS. Afterwards, the sections were incubated with the following secondary antibody: goat anti-rabbit Alexa 488 (1:1000, 111-545-003) in 5% NGS in PBS for 2 h at room temperature. Finally, the sections were rinsed 4 times for 5 min with PBS at room temperature and mounted onto slides using Fluoromount-G (0100-20). Sections containing the CA3 region were imaged using a Zeiss confocal at 10× magnification. Each confocal image was obtained using identical settings for gain, laser and pinhole size. The images were then analyzed using ImageJ FIJI software (https://imagej.net/software/fiji/downloads, accessed on 2 August 2022; version: 2.1.0/153c, Build: 5f23140693, Open source image processing software, copyright: 2010–2022).

Note that affinity-purified rabbit polyclonal OCT3 primary antibodies were generated against a 19 aa C-terminal cytoplasmic domain of mouse OCT3. This peptide has no significant sequence homology with other mouse OCTs. Preabsorption immunohistochemistry (IHC) staining experiments were performed in which hippocampal sections were incubated in buffer containing the normal working concentration of primary antibody (OCT3 or PMAT) with a 5x excess of control antigen. No nonspecific staining was observed. Two additional control experiments were performed. First, tissue sections were incubated in primary OCT3 or PMAT antibody, but no secondary antibody was used. Second, tissue sections were stained as normal but nonimmune rabbit IgG was used in place of primary OCT3 or PMAT antibody. In both cases, no nonspecific staining was observed.

### 2.7. Drugs

Decynium-22 (D22) [1,1′-Diethyl-2,2′-cyanine iodide] (Sigma-Aldrich, St. Louis, MO, USA) was dissolved in physiologic saline and injected i.p. at a dose expressed as salt weight per kilogram body weight. The dose of D22 (0.1 mg/kg) was selected based on our previous studies in adults showing antidepressant-like effects when SERT is either pharmacologically [15] or genetically [16] compromised, while not influencing general locomotor activity.

### 2.8. Data Analysis

Data were analyzed using GraphPad Prism version 6.0 (San Diego, CA, USA). Data are expressed as mean ± S.E.M. P ≤ 0.05 was considered statistically significant for all analyses.

TST data comparing P21 and P90 mice after receiving either saline or 0.1 mg/kg D22 were analyzed using a two-way ANOVA (age × drug treatment) followed by Tukey’s post-hoc multiple comparisons test.

To examine serotonin clearance kinetics in P21 and P90 mice, data were fit to the Michaelis–Menten equation to calculate apparent maximal velocity (V_max_) for serotonin clearance and the apparent transporter affinity (K_m_, but denoted as K_T_ here to make clear that this is an affinity value determined in vivo, so diffusion is a factor). Serotonin clearance rate was analyzed by a two-way repeated measures ANOVA (age x serotonin concentration) with Bonferroni’s post-hoc multiple comparisons. Bonferroni’s method was used since it does not assume independence [30]. Comparison of P21 and P90 serotonin signal parameters (T_c_, T_20_–T_60_, and T_80_) at signals yielding peak amplitudes of ~1.0 µM serotonin were analyzed using unpaired *t*-tests.

The number of neurons and microglia in CA3 region of hippocampus of P21 and P90 mice were analyzed by two-way ANOVA (age × cell type) followed by Tukey’s post-hoc multiple comparisons test. The area of CA3 region of hippocampus of P21 and P90 mice was analyzed by unpaired *t*-tests.

Western blot density (normalized to β-actin) and for IHC, intensity of fluorescence in CA3 region of hippocampus, of P21 and P90 mice were analyzed using unpaired *t*-tests.

## 3. Results

### 3.1. D22 Produces an Antidepressant-like Effect in Juvenile, but Not Adult, Mice

**Figure 1:**
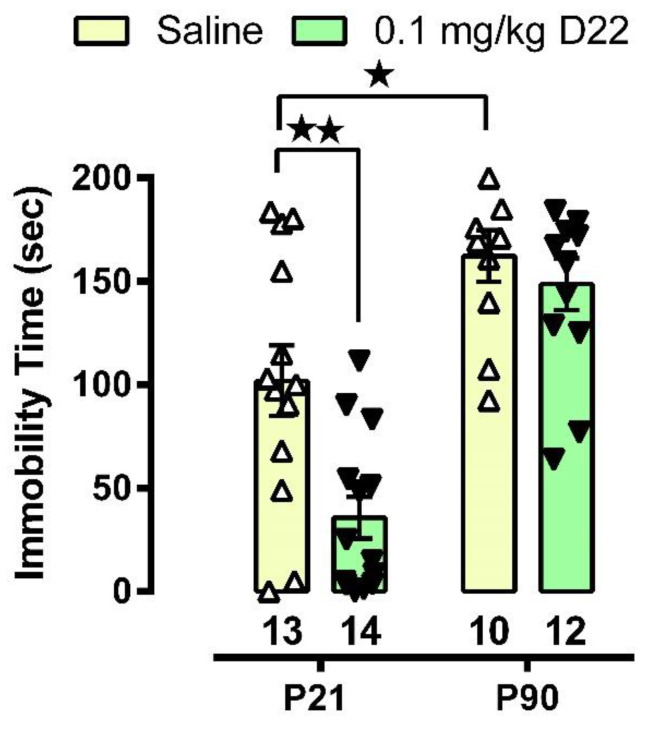
Blockade of OCTs and PMAT with D22 produces an antidepressant-like effect in juvenile (P21) mice but not in adult (P90) mice. Immobility time (s) in the TST after acutely administered saline (open bars, open triangles) or D22 (green bars, filled inverted triangles). Tukey’s post hoc test for multiple comparisons after a two-factor ANOVA. * *p* < 0.05 and ** *p* < 0.01 significant difference from vehicle. Data are mean ± S.E.M. Sample size is indicated under each bar.

As shown in Figure 1, juvenile mice, regardless of treatment, displayed shorter immobility times than adult mice. Two-way ANOVA revealed a significant main effect of age [F(1,45) = 41.24, *p* < 0.0001] and treatment [F(1,45) = 8.725, *p* = 0.005], with an interaction approaching significance [F(1,45) = 3.805, *p* = 0.057]. Replicating our previous work [31], Tukey’s post-hoc analyses showed that juvenile mice injected with saline spend less time immobile in the TST than adult mice (*p* = 0.019). Also consistent with our earlier work [15,16], D22 (0.1 mg/kg, i.p) had no standalone antidepressant-like effect (i.e., ability to reduce immobility time) in adult, wildtype mice (*p* = 0.906). Extending these findings, and in contrast to adults, we found that D22 does have a standalone antidepressant-like effect in juvenile mice (*p* = 0.004). These findings suggest that the antidepressant-like effect of D22 in juveniles is driven by its action at OCTs and/or PMAT.

### 3.2. Juvenile Mice Clear Serotonin from Extracellular Fluid Faster than Adult Mice

**Figure 2:**
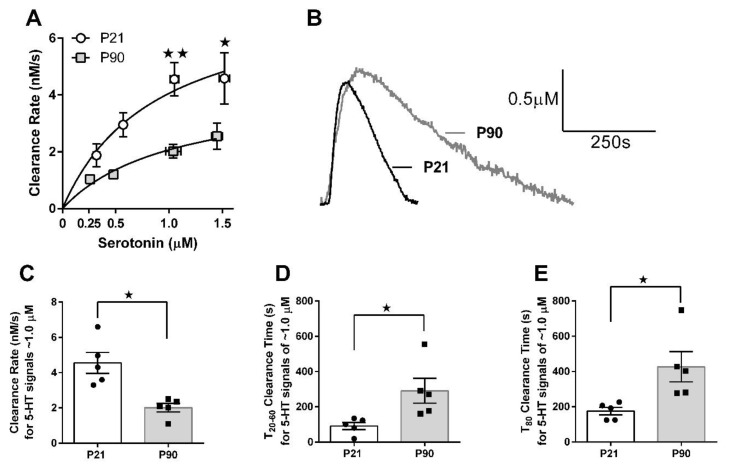
Juvenile mice clear serotonin faster from extracellular fluid in CA3 region of hippocampus than adult mice. (**A**) Serotonin clearance rate as a function of increasing extracellular serotonin concentration. Serotonin was intrahippocampally applied to obtain signals with amplitudes of approximately 0.25, 0.50, 1.0 and 1.5 µM. Horizontal error bars represent variance (S.E.M) in signal amplitudes achieved. Vertical error bars are S.E.M. for clearance rate. Where error bars are not visible, they are obscured by the symbol. (**B**) Representative traces of serotonin clearance in juvenile (P21, circles) and adult (P90, squares) mice for signal amplitudes of ~1.0 µM. Summary data for serotonin clearance rate (nM/s) (**C**), T_20–60_ clearance time (s) (**D**) and T_80_ clearance time (s) (**E**) for signal amplitudes of ~1.0 µM. (**A**) * *p* < 0.05, ** *p* < 0.01 P21 significantly different from P90 mice, 2-way repeated measures ANOVA with Bonferroni’s post-hoc comparisons. (**C**–**E**) * *p* < 0.05, unpaired *t*-tests with Welch’s correction for significantly different variances in D and E. Data are mean ± S.E.M. *n* = 5 per group.

In vivo high-speed chronoamperometry was used to examine serotonin clearance kinetics in the CA3 region of the hippocampus of P21 and P90 mice (Figure 2). Representative traces of juvenile and adult serotonin signals at a concentration of ~1.0 µM are shown in Figure 2B. A repeated measures two-way ANOVA (age × concentration) revealed a significant main effect of serotonin concentration [F(3,24) = 13.15, *p* < 0.0001], a significant effect of age [F(1,24) = 14.62, *p* = 0.005], but no significant interaction [F(3,24) = 1.679, *p* = 0.198]. As per recommendations [32,33], post hoc analyses were conducted even though the interaction was not statistically significant at the 5% level. Post hoc analyses showed juvenile mice to have a significantly faster serotonin clearance rate at 1.0 µM serotonin (*p* = 0.01) and 1.5 µM serotonin (*p* = 0.05; Figure 2A) than adult mice. Apparent maximal velocity (V_max_) and affinity (denoted K_T_ to distinguish this value as transporter affinity as measured in vivo, where diffusion factors) of serotonin clearance were derived using the Michaelis–Menten equation. For juvenile mice V_max_ = 7.64 ± 2.56 nM/s and K_T_ = 0.88 ± 0.62 µM, and for adult mice V_max_ = 4.25 ± 1.42 nM/s and K_T_ = 1.06 ± 0.68 µM. Faster rates of serotonin clearance in juvenile mice were, not surprisingly, also reflected by shorter clearance time (T_20_–T_60_ and T_80_). For example, at serotonin signal amplitudes of ~1.0 µM clearance rate differed significantly between juvenile and adult mice [t(8) = 3.99, *p* = 0.004, Figure 2C], as did T_20_–T_60_ [t(4.659) = 2.72, *p* = 0.05. Figure 2D] and T_80_ [t(4.488) = 2.85, *p* = 0.04, Figure 2E], with Welch’s correction for significantly different variances between groups (Figure 2D,E).

Of note, in a pilot study, we showed that one hour following D22 (0.1 mg/kg i.p., i.e., the same conditions as used in TST studies) serotonin clearance from extracellular fluid in CA3 region of hippocampus of juvenile mice was inhibited (49 ± 19% increase in T_80_ clearance time, *n* = 3). This is consistent with our finding that this dose of D22 produced a standalone antidepressant effect in juvenile mice (Figure 1), but did not in adults, nor did it inhibit serotonin clearance in adult mice (see [15,16]).

### 3.3. Density of Neurons and Microglia in CA3 Region of Hippocampus Does Not Differ between Juvenile and Adult Mice

**Figure 3:**
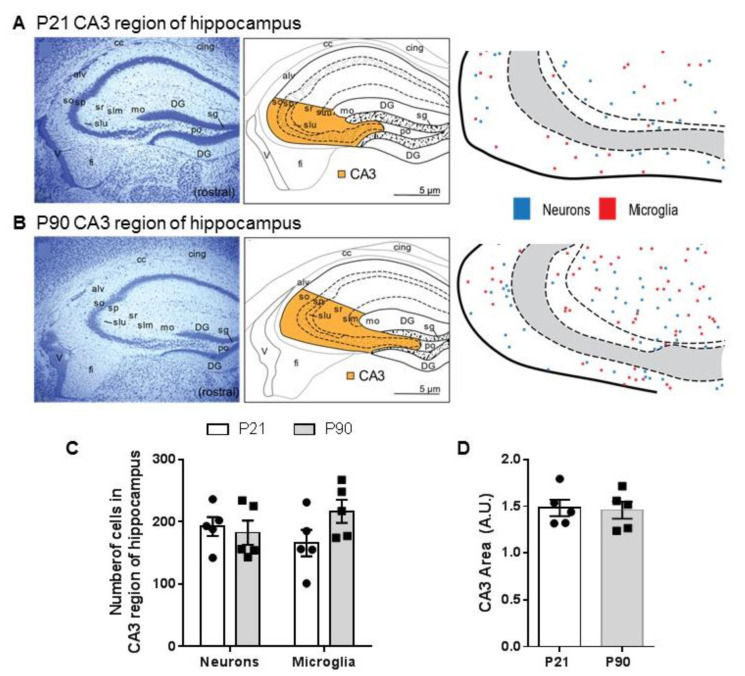
Juvenile and adult mice have a comparable number of neurons and microglia in CA3 region of hippocampus. Representative section of hippocampus with thionin stain, accompanied by maps from Allen Brain Atlas highlighting CA3 region, and traces of CA3 region with marks indicating location of neurons and microglia in (**A**) juvenile mice (P21) and (**B**) adult mice (P90). (**C**) Summary data showing no difference in the number of neurons and microglia in CA3 region of hippocampus between juvenile and adult mice. (**D**) Summary data showing no difference in the total area of CA3 region of hippocampus between juvenile and adult mice. Data are mean ± S.E.M. *n* = 5/group (same mice as used in chronoamperometry recordings). Abbreviations: alv alveus; cc corpus callosum; cing cingulum; DG dentate gyrus; fi fimbria; mo molecular layer; po polymorph layer; sg granular layer; slm stratum lancunosum moleculare; slu stratum lucidem; so stratum oriens; sp pyramidal layer; sr stratum radiatum; v lateral ventricle.

To determine whether enhanced serotonin clearance in juvenile mice could be due to reduced tortuosity (i.e., allowing serotonin to diffuse more quickly through the extracellular matrix rather than as a function of increased transporter capacity), we examined the number of cells in CA3 region of hippocampus in juvenile and adult mice. We found no significant difference in the number of cells in this region between P21 and P90 mice [t(6) = 0.002, *p* = 0.99], nor in their distribution (Figure 3A–C). To obtain information about cell type, we analyzed the number of neurons and microglia. Two way ANOVA (age, cell type) did not show statistically significant main or interaction effects [F(1,16) = 2.638 *p* = 0.1239] (Figure 3). We also analyzed the area of the CA3 region of hippocampus between juvenile and adult mice to determine if any differences in size exist that may contribute to cell density. We found no statistically significant difference in the size of CA3 region of hippocampus between juvenile and adult mice [t(8) = 0.18, *p* = 0.86]. 

### 3.4. Greater PMAT, but Not OCT3, Expression in Juvenile Hippocampus than in Adult

**Figure 4:**
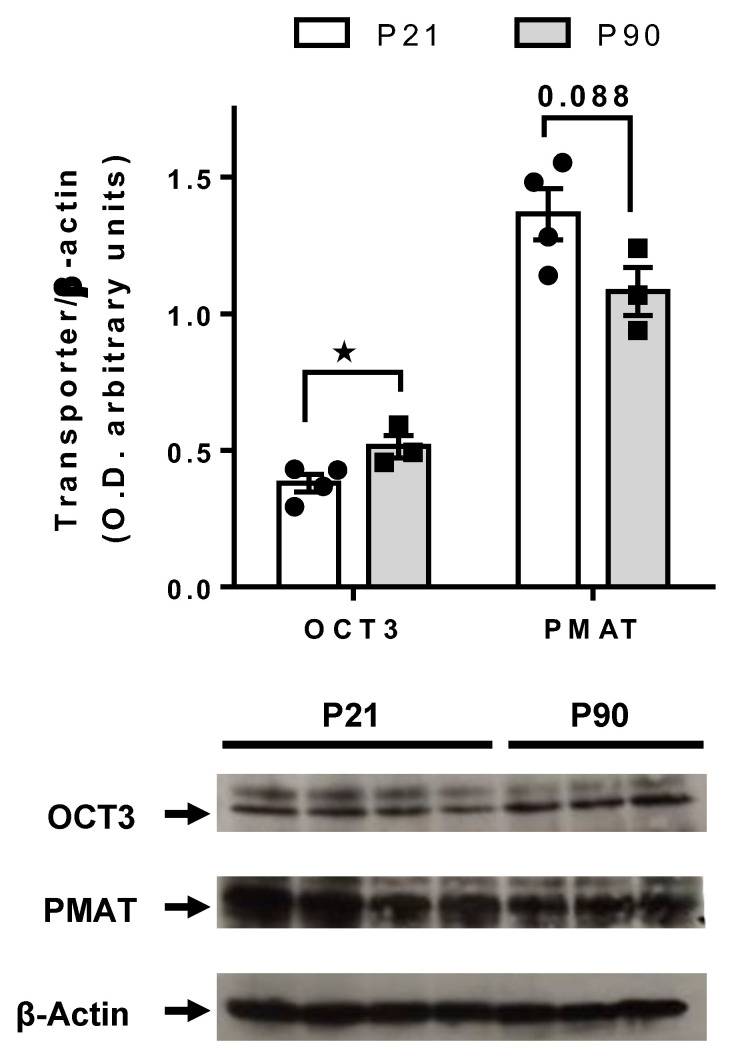
Western blot analyses revealed a trend for greater hippocampal PMAT expression, and lower OCT3 expression, in juvenile mice than in adults. Bands for OCT3, PMAT and β-Actin corresponded to the expected molecular weights of 70, 58 and 40 kDa, respectively. * *p* < 0.05, unpaired *t*-tests. Data are mean ± S.E.M. P21 *n* = 4, P90 *n* = 3.

As shown in Figure 4, using PMAT and OCT3 specific antibodies, western blot analyses revealed a trend for greater PMAT expression in the hippocampus of juvenile mice than adults [t(5) = 2.118, *p* = 0.088], whereas hippocampal OCT3 expression was significantly greater in adult mice than juveniles [t(5) = 2.585, *p* = 0.049]. Juvenile mice had 26% more PMAT, and 26% less OCT3 in hippocampus relative to adult mice.

**Figure 5:**
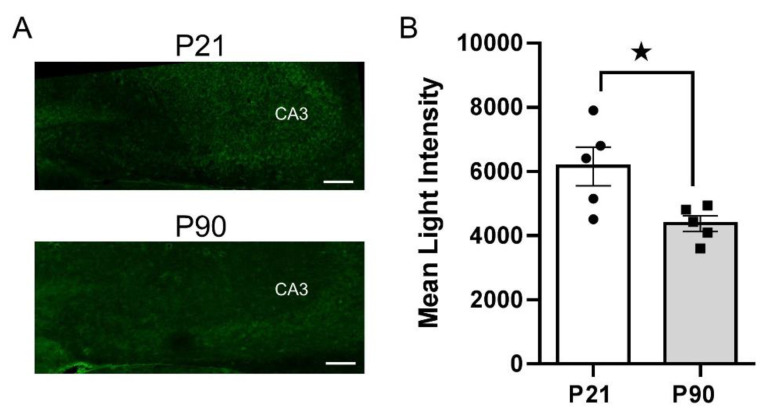
Juvenile mice express higher levels of PMAT in CA3 region of hippocampus. (**A**) Representative 10× images from juvenile (P21) (top) and adult (P90) (bottom) mice. The mean intensity of light measurements was taken from the same area as that indicated in cell count data (Figure 3). (**B**) Summary data showing the difference in PMAT expression between juvenile (open bar, circles) and adult (grey bar, squares) mice. * *p* < 0.05, unpaired t-test for independent samples. Data are mean ± S.E.M. *n* = 5 per age. Scale bar = 100 µM.

**Figure 6:**
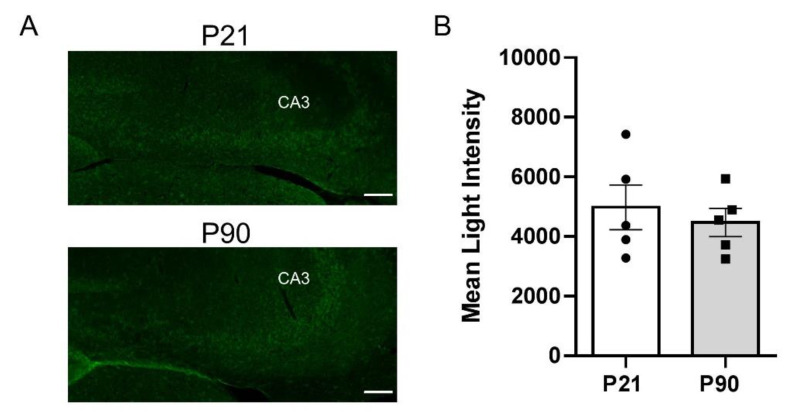
OCT3 expression in CA3 region of hippocampus is similar between juvenile and adult mice. (**A**) Representative 10× images from juvenile (P21) (top) and adult (P90) (bottom) mice. The mean intensity of light measurements was taken from the same area as that indicated in cell count data (Figure 3). (**B**) Summary data showing no difference in OCT3 expression between juvenile (open bar, circles) and adult (grey bars, squares) mice. Data are mean ± S.E.M. *n* = 5 per age. Scale bar = 100 µM.

To gain more precise anatomical resolution, we turned to IHC to label PMAT and OCT3 with specific antibodies and quantify expression in CA3 region of hippocampus by measuring mean light intensity. Consistent with western blot analyses on whole hippocampal homogenates, we found significantly greater PMAT expression in CA3 region of hippocampus (the region where chronoamperometric recordings were made) in juvenile mice (6158 ± 1346) compared to adults (4375 ± 543) [t(8) = 2.746, *p* = 0.025] (Figure 5). However, there was no significant difference in OCT3 expression between juvenile (4978 ± 1682) and adult (4469 ± 1051) mice [t(8) = 0.574, *p* = 0.582], suggesting that greater OCT3 expression in adult mice detected via western blot likely results from heightened expression in hippocampal sub-regions other than CA3 region (Figure 6).

## 4. Discussion

The main findings from these studies are that the OCT/PMAT blocker D22 has an antidepressant-like effect in juvenile, but not in adult mice (Figure 1), and that juvenile mice clear serotonin from extracellular space in the CA3 region of hippocampus faster than adult mice (Figure 2). Faster serotonin clearance in juvenile hippocampus does not appear to be related to reduced tortuosity, as cell density and size of CA3 region did not differ between juvenile and adult mice (Figure 3). Given that juvenile mice have fewer SERT in a broad range of brain regions, including CA3 region of hippocampus [34], and evidence that the affinity of SERT is reduced in juvenile hippocampus [31], these data suggest that faster serotonin clearance in juvenile mice is due to functionally upregulated non-SERT serotonin transporters, putatively OCT3 and/or PMAT, which are low-affinity, high-capacity transporters for serotonin. Western blot analyses of whole hippocampal homogenates showed that PMAT expression trended to be higher in juvenile mice than in adults, while OCT3 expression was lower in juvenile than adult mice (Figure 4). IHC quantification of PMAT and OCT3 in CA3 region of hippocampus showed that PMAT expression was greater in juvenile mice than adults, whereas OCT3 expression was not different between the two ages (Figure 5 and Figure 6). That serotonin clearance rate was significantly faster only at higher serotonin concentrations (i.e., 1.0 and 1.5 µM, Figure 2) lends support to the idea that the low-affinity, high-capacity serotonin transporter, PMAT, is at least in part responsible for faster serotonin clearance rates in juvenile mice. Importantly, faster serotonin clearance in juveniles by non-SERT transporters, putatively PMAT, provides a mechanistic basis for poorer therapeutic efficacy of SSRIs in children than in adults [1].

We did not evaluate a role for OCT1 in this study because its expression in brain is relatively low, monoamines are generally reported to be poor substrates for this transporter, and D22 has lower affinity for OCT1 than other OCTs or PMAT [18,35]. D22 has been reported to have lower affinity for OCT2 than OCT3 or PMAT (e.g., [19]), although other studies show it to have equivalent affinity (see [18]); thus, we cannot rule out a possible role for OCT2 in our present findings.

Since volume fraction of extracellular space (alpha) and tortuosity (lambda) can impact the diffusion of neurotransmitter through the extracellular matrix [24,36,37,38], it was important to quantify cell density to determine if lower cell density in juvenile mice may contribute to faster serotonin clearance. We found no statistically significant differences in cell counts between juvenile and adult mice, including no differences in the number of neurons or microglia between juvenile and adult mice, nor in the size of the CA3 region of hippocampus. Our data corroborate Lehmenkühler and colleagues [36], who found that there was no difference in volume fraction between P21 and P90-120 mice. Thus, it appears unlikely that increased volume fraction or reduced tortuosity accounts for faster serotonin clearance in juvenile mice.

Consistent with a prominent role for low-affinity, high-capacity transporters in clearance of serotonin in juvenile mice, serotonin clearance was only significantly faster in juvenile mice at concentrations of serotonin 1.0 μM and higher (i.e., OCTs/PMAT recruiting concentrations). Together with our previous findings that SERT expression, and likely function, is less in juvenile than adult mice [31,34] our data support the idea that low-affinity, high-capacity transporters, putatively PMAT, are key players in serotonin clearance in juvenile mice. Both DAT and NET are low-affinity, high-capacity transporters for serotonin [39,40,41,42,43,44,45]. Given the sparse expression of DAT in hippocampus [46,47], it is unlikely that DAT accounts for faster serotonin clearance in juvenile mice. NET is widely expressed in hippocampus, including the CA3 region; however, we found that juvenile mice have fewer NETs in CA3 region of hippocampus, as well as other terminal fields compared to adults [48]. Moreover, affinity for [^3^H]nisoxetine binding to NET did not differ between juvenile and adult mice [48]. Taken together, our findings suggest that NET is also unlikely to account for faster serotonin clearance in juvenile mice.

Because our studies in adult mice point to D22-sensitive transporters, putatively OCT3 and/or PMAT as important regulators of serotonin clearance, particularly when SERT is pharmacologically or genetically compromised [15,16], we hypothesized that these transporters may account for faster serotonin clearance in juvenile mice. Our western blot and IHC results suggest faster serotonin clearance in juveniles is due to having higher levels of PMAT than adults. In contrast, expression of OCT3 in whole hippocampal homogenates was lower in juveniles than adults, and in CA3 region of hippocampus, where chronoamperometry recordings were made, it did not differ between ages. Given that PMAT is the most efficient transporter of serotonin of the D22-sensitive transporters [13], our results align with the possibility that PMAT is a key driver of serotonin clearance in juveniles.

Given that SERT expression and function is less in juvenile mice than in adults [31,34], and assuming D22-sensitive transporters, putatively PMAT based on our results, are responsible for the lion’s share of serotonin clearance in juveniles, then blockade of these transporters would be expected to elicit antidepressant-like effects. Consistent with this idea, we found that D22 produced a standalone antidepressant-like effect in juvenile mice, but not in adults. Of note, in parallel with clinical literature, we previously found that the antidepressant-like response of juvenile mice to the SSRI escitalopram is dampened compared to adult mice [31,34]. Thus, the present findings suggest that functionally upregulated D22-sensitive transporters, putatively PMAT, may account, at least in part, for reduced antidepressant efficacy of SSRIs in juveniles.

## 5. Conclusions

Together, the present results encourage further investigation into the role of D22-sensitive transporters, putatively PMAT, in serotonergic neurotransmission and antidepressant response in juveniles. Furthermore, the therapeutic efficacy of SSRIs is also less in adolescents than in adults, warranting future investigations into the contribution of D22-sensitive transporters to serotonergic homeostasis in adolescents as well. Finally, it should be kept in mind that D22-sensitive transporters are capable of transporting monoamines other than serotonin, albeit with varying affinities (for reviews see [11,12,18,35]). Thus, blockade of these transporters would be expected to increase extracellular levels of all monoamines. Given clinical reports that antidepressants that block SERT, NET and DAT are therapeutically more efficacious than those that block only one or two of these transporters [49], blockade of PMAT, and potentially other D22-sensitive transporters such as OCT2 and OCT3, are deserving of further study as a useful therapeutic strategy, either as a standalone antidepressant (putatively in juveniles) or as an adjunctive treatment to existing antidepressants for sufferers of depression of all ages.
